# HOXC11–SRC-1 regulation of S100beta in cutaneous melanoma: new targets for the kinase inhibitor dasatinib

**DOI:** 10.1038/bjc.2011.193

**Published:** 2011-06-07

**Authors:** C deBlacam, C Byrne, E Hughes, M McIlroy, F Bane, A D K Hill, L S Young

**Affiliations:** 1 Endocrine Oncology Research, Department of Surgery, Royal College of Surgeons in Ireland, York House, York Street, Dublin 2, Ireland

**Keywords:** HOXC11, SRC-1, S100beta, melanoma, dasatinib

## Abstract

**Background:**

Cutaneous melanoma is an aggressive disease. S100beta is an established biomarker of disease progression; however, the mechanism of its regulation in melanoma is undefined.

**Methods:**

Expression of HOXC11 and SRC-1 was examined by immunohistochemistry and immunofluorescence. Molecular and cellular techniques were used to investigate regulation of S100beta, including, western blot, qPCR, ChIP and migration assays.

**Results:**

Expression levels of the transcription factor HOXC11 and its coactivator SRC-1 were significantly elevated in malignant melanoma in comparison with benign nevi (*P*<0.001 and *P*=0.017, respectively, *n*=80), and expression of HOXC11 and SRC-1 in the malignant tissue associated with each other (*P*<0.001). HOXC11 recruitment to the promoter of S100beta was observed in the primary melanoma cell line SKMel28. S100beta expression was found to be dependant on both HOXC11 and SRC-1. Treatment with the Src/Abl inhibitor, dasatinib, reduced HOXC11–SRC-1 interaction and prevented recruitment of HOXC11 to the S100beta promoter. Dasatinib inhibited both mRNA and protein levels of S100beta and reduced migration of the metastatic cell line MeWo.

**Conclusion:**

We have defined a signalling mechanism regulating S100beta in melanoma, which can be modulated by dasatinib. Profiling patients for expression of key markers of this network has the potential to increase the efficacy of dasatinib treatment.

Classic pathologic parameters such as tumour depth and lymph node status are routinely used to predict clinical outcome in cutaneous melanoma. However, a significant number of accurately staged patients will develop an unpredictable pattern of disease recurrence. With the current dearth of effective treatments for advanced melanoma, it is increasingly recognised that elucidation of the molecular mechanisms of tumour progression will inform individualised therapy. Recently, serum levels of the calcium-binding protein S100beta have been used as a marker of tumour burden, response to treatment and prognosis. Though no consensus exists on its implementation in the routine clinical setting, recent meta analysis suggests that serum S100beta detection has clinical value as an independent prognostic marker in melanoma patients (Mocellin *et al*, 2008).

S100beta is a member of the S100 family, it can act as a stimulator of proliferation and migration and an inhibitor of apoptosis and differentiation (reviewed in Donato *et al*, 2009). A mechanism by which activation of S100beta occurs is unclear. A variety of signals modulates its release and/or expression, including interferon gamma, interleukins, fibroblast growth factor-2, glutamate, corticosterone and pregnisone (Modi and Kanungo, 2010). In neuronal cells, forced expression of HOXC6 and HOXC11 has been shown to induce S100beta expression (Zhang *et al*, 2007). More recently, in breast cancer, we have reported a role for HOXC11 in conjunction with the transcriptional activator SRC-1, in the regulation of S100beta (McIlroy *et al*, 2010). Aberrant expression of SRC proteins has been associated with a more aggressive phenotype in a number of cancers including breast, thyroid and prostate. (Gregory *et al*, 2001; Redmond *et al*, 2009; Kavanagh *et al*, 2010). Recent studies provide evidence of a specific role for SRC-1 in the development of metastases (Wang *et al*, 2009). Activation of SRC-1 is through Src kinase, suggesting that this is a signalling network that could be targeted by the ATP-competitive dual Src/Abl inhibitor, dasatinib. Dasatinib is used in the treatment of chronic myeloid leukaemia and is currently in trials for the treatment of advanced melanoma (http://www.clinicaltrials.gov). Though data from these trials are emerging only now, early analysis suggests that patient selection with accurate biomarkers would have the potential to dramatically improve response rates (Kluger *et al*, 2010).

A full understanding of the signalling mechanisms that are targeted by emerging treatments will uncover biomarkers to enable careful patient selection and provide new targets in this class. In this study, we describe the transcriptional regulation of S100beta in melanoma and show efficacy for dasatinib in disrupting this signalling network.

## Materials and methods

### Cell culture

SKMel28 cells, derived from a primary cutaneous melanoma, and MeWo cells, derived from a melanoma lymph node metastasis, were obtained from ATCC and were maintained in Eagle's minimal essential medium (MEM) (Invitrogen, CA, USA) supplemented with 2 mM L-glutamine, 100 *μ*g ml^−1^ penicillin, 100 *μ*g ml^−1^ streptomycin (Sigma-Aldrich, Ireland Ltd, Wicklow, Ireland) and 10% (v/v) FBS (Gibco, Invitrogen). Primary cell cultures were prepared from patients with malignant melanoma. Melanoma cells were dissociated from the tumour mass using a digestion mixture (DMEM-F12, 10% FBS, 10 *μ*g ml^−1^ insulin, 100 U ml^−1^ hyaluronidases and 200 U ml^−1^ collagenases) and were cultured as above, *n*=4.

### Immunofluorescence

Following ethical approval from Beaumont Hospital, malignant melanoma and benign nevi tissue were collected and embedded in paraffin. Immunofluorescent staining on paraffin embedded tissue and primary cultures was performed using chicken anti-HOXC11 (0.4 *μ*g ml^−1^) and rabbit anti-SRC-1 (0.5 *μ*g ml^−1^), followed by the corresponding fluorescent-conjugated antibodies FITC anti-chicken and TRITC anti-rabbit. Nuclei were counterstained with 4′,6-diamidino-2-phenylindole. Staining was visualised using a confocal microscope.

### Coimmunoprecipitation and western blot

SKMel 28 and MeWo cell lines were treated with either vehicle or dasatinib (100 nM) for 2 h. Protein (1 *μ*g) was extracted and immunoprecipitated with rabbit anti-SRC-1 (SC-8995; Santa Cruz Biotechnology, Inc., Santa Cruz, CA, USA). Resultant proteins were subjected to SDS–PAGE and subsequently probed with either rabbit anti-SRC-1 (SC-8995; Santa Cruz) or chicken anti-HOXC11 (15-288-22000F; GenWay Biotech, Inc., San Diego, CA, USA). HSP70 or *β*-actin was used as a loading control.

### Overexpression and knockdown of HOXC11

SKMel28 cells were transfected with control vector pDEST47 (400 ng) or pDEST47 HOXC11 (400 ng). Protein levels were determined 24 h post transfection by western blot. Predesigned and validated siRNA directed against HOXC11 (Qiagen Ltd, Crawley, West Sussex, UK) and SRC-1 (Applied Biosystems/Ambion, Warrington, UK) was used in the knockdown studies. Protein and mRNA levels were assessed 24 h post-transfection.

### PCR

Relative mRNA levels of S100beta were determined as described (McIlroy *et al*, 2010).

### ChIP

ChIP was performed as previously described (McIlroy *et al*, 2010). Cells were treated with either vehicle or dasatinib (100 nM) for 2 h. Chicken anti-HOXC11 (7 *μ*g), or H4 antibody (7 *μ*g), was added to the supernatant fraction of SKMel 28 cells and incubated overnight at 4°C with rotation. Proteins were un-crosslinked and primers were used to amplify the DNA −424 to +24 of the S100beta transcriptional start site.

### Immunohistochemistry

Immunohistochemical staining for S100beta was performed using mouse anti-S100beta (Ab8330; Abcam, Cambridge, UK, 2 *μ*g ml^−1^) followed by the corresponding antibody; in benign naevus and malignant melanoma; tissue was counterstained with haematoxylin. Images are representative of 64 separate melanoma tumours and 20 benign nevi.

### Migration assay

Cellomics Cell Motility Kit (Thermo Scientific/Fisher Scientific Ireland, Dublin, Ireland, #K0800011) was used to assess individual cell movement after 22 h. In short, 96-well plates were precoated with collagen. Cells were seeded at 1 × 10^4^ cells ml^−1^ on a lawn of blue fluorescent beads. The track area is proportional to the magnitude of cell movement. Track areas were quantitatively analysed using Olympus cell^F^ imaging software. Student t-test was used to compare mean migratory track areas. MeWo cells were treated with vehicle or dasatinib (100 nM) and migration was assessed using the standard scratch assay.

### Proliferation assay

2000 SKMel28 and MeWo cells were plated in a 96-well plate and incubated overnight at 37°C and then treated with dasatinib (1 and 10 *μ*M). Proliferation was assayed 72 h later by MTS CellTiter 96 (Promega, Madison, WI, USA) following the manufacturer's instructions.

## Results

### HOXC11 and SRC-1 in malignant melanoma

**Figure 1:**
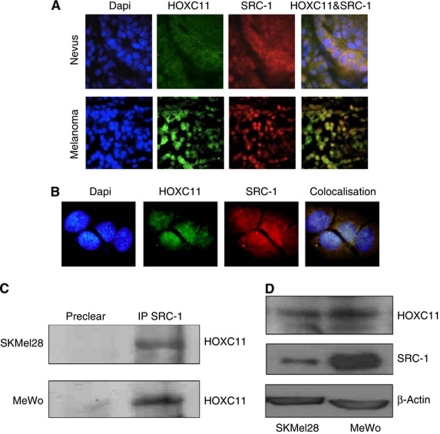
Immunofluorescent staining of HOXC11 and SRC-1 in (**A**) benign nevus and malignant melanoma tissue, × 100, and (**B**) in primary cell cultures derived from melanoma patient tissue, × 200. (**C**) Interaction of HOXC11 and SRC-1 in melanoma cell lines shown by immunoprecipitation. (**D**) Expression levels of HOXC11 and SRC-1 in primary (SKMel28) and metastatic (MeWo) melanoma cell lines by western blot. All blots are representative of three separate experiments.

Combined immunohistochemical and immunofluorescence was used to assess the expression of HOXC11 and its coactivator protein SRC-1 in benign nevi and malignant melanoma. In tissue from 80 patients assessed, nuclear expression of both HOXC11 and SRC-1 was significantly expressed more frequently in malignant melanoma in comparison with benign nevi (*P*<0.001 and *P*=0.017, respectively), Table 1. Moreover, expression of HOXC11 and SRC-1 in the malignant tissue is significantly associated with each other (*P*<0.001, Fisher's exact test). HOXC11 and SRC-1 were expressed predominantly in cytoplasm of cells in the nevi with no detectable nuclear coassociation (Figure 1A). In the tumour tissue and in primary cell cultures derived from patient tumours, both HOXC11 and SRC-1 translocate to the nucleus with a high level of coassociation (Figures 1A and B). Immunoprecipitation studies confirmed HOXC11 and SRC-1 interaction in primary (SKMel28) and malignant (MeWo) melanoma cell lines (Figure 1C). Greater protein expression of both HOXC11 and SRC-1 were found in metastatic melanoma cells in comparison with primary melanoma cell lines (Figure 1D).

### HOXC11 and SRC-1 cooperate to regulate S100beta in melanoma cells

**Figure 2:**
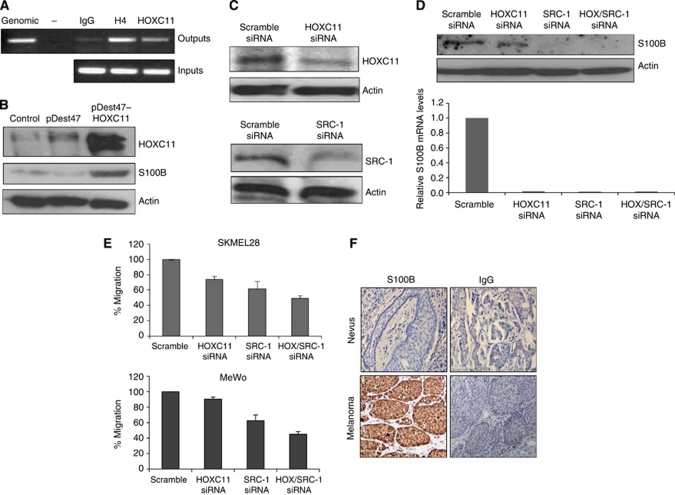
HOXC11 and SRC-1 cooperate to regulate S100beta in melanoma cells. (**A**) ChIP analysis of HOXC11 recruitment to the S100beta promoter region in SKMel28 cells. Genomic DNA, no template control, IgG and H4 control samples are shown. (**B**) Overexpression of HOXC11 in SKMel28 cells (pDEST47 HOXC11) upregulated protein expression of S100beta in comparison with control (pDEST47). (**C**) Confirmation of knockdown by western blot. SKMel28 cells were transfected with siRNA HOXC11, siRNA SRC-1 or scrambled siRNA. (**D**) Knockdown of HOXC11 and SRC-1 inhibits mRNA and protein expression of S100beta. Results are representative of three separate experiments. (**E**) Combined knockdown of HOXC11 and SRC-1 results in a significant decrease in migration in both SKMel28 and MeWo melanoma cells (*P*<0.05, Student's *t*-test). Results are expressed as mean±s.e.m. (*n*=3). (**F**) Immunohistochemical localisation of S100beta in benign naevus and malignant melanoma, × 100. Images are representative of 64 separate melanoma tumours and 20 benign nevi.

Using chromatin immunoprecipitation, we established HOXC11 recruitment to the promoter of S100beta in primary melanoma cells (Figure 2A). Forced expression of HOXC11 enhanced protein levels of S100beta in the SKMel28 cells (Figure 2B). Furthermore, knockdown of either HOXC11 alone or in combination with SRC-1 significantly reduced S100beta expression at both the RNA and protein level (Figures 2C and D). In addition, combined knockdown of HOXC11 and SRC-1 results in significantly decreased migration of both SKMel28 and MeWo (Figure 2E). In line with our observation of increased expression and interaction between HOXC11 and SRC-1 in malignant melanoma versus benign nevi, high expression levels of their putative target S100beta were detected in melanoma tissue in comparison with little or no expression in the naevus tissue (Figure 2F).

### Dasatinib can inhibit S100beta production in melanoma

**Figure 3:**
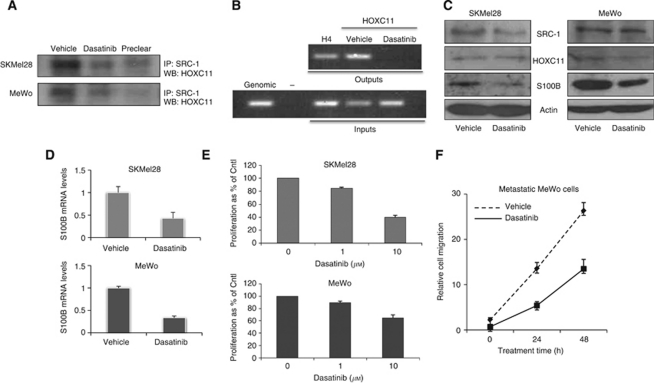
Effect of Src kinase inhibition with dasatinib on HOXC11–SRC-1 S100beta network in melanoma cells. (**A**) Treatment of SKMel28 and MeWo cells with dasatinib (100 nM) reduces HOXC11–SRC-1 interactions. SRC-1 was immunoprecipated from SKMel28 and MeWo cells and immunoblotted for HOXC11. (**B**) Src kinase inhibition reduces HOXC11 recruitment to the S100beta promoter. (**C**) Treatment with dasatinib reduces S100beta protein expression in SKMel28 and MeWo cells. Little or no alteration in protein expression of SRC-1 or HOXC11 was detected. All blots are representative of three separate experiments. (**D**) Treatment of SKMel28 and MeWo cells with dasatinib reduces S100beta expression at the transcriptional level. (**E**) Treatment with dasatinib at 10 *μ*M, but not 1 *μ*M, reduced cell proliferation in both cell lines, (*P*<0.05, Student's *t*-test). (**F**) Dasatinib (100 nM) reduces migration in MeWo malignant melanoma cells (*P*<0.05, ANOVA). All results are expressed as mean±s.e.m. (*n*=3).

We investigated whether inhibition of the Src kinase signalling network could have a role in disrupting the functional interaction between HOXC11 and SRC-1 in melanoma cells. The ATP-competitive dual Src/Abl inhibitor dasatinib reduced HOXC11–SRC-1 interaction and prevented recruitment of HOXC11 to the promoter region of S100beta (Figures 3A and B). Dasatinib had little or no effect on the expression levels of SRC-1 or HOXC11 in either SKMeL28 or MeWo cells (Figure 3C). Treatment with dasatinib significantly reduced both protein and RNA expression of S100beta in melanoma cell lines (Figures 3C and D). High levels of dasatinib were required to reduce cell proliferation in both cell lines (Figure 3E). In the metastatic melanoma cell line MeWo, however, treatment with dasatinib at 100 nM was sufficient to reduce cell migration over time (Figure 3F).

## Discussion

Cutaneous melanoma is characterised by an unpredictable disease course. Studies now suggest that a particular signature emerges during the vertical growth phase of the primary tumour that can mark the emergence of the metastatic phenotype. Work from Riker *et al* (2008) has found that the homeobox family of proteins are among a group of six oncogenes that alter expression during the metastatic transition. Aberrant homeobox gene expression has been shown to promote tumourigenesis through a variety of mechanisms (Samuel and Naora, 2005; Maeda *et al*, 2005; Shah and Sukumar, 2010). The role of the homeobox genes in the transcriptional regulation of S100beta in breast tumour and neuronal tissue (Zhang *et al*, 2007; McIlroy *et al*, 2010) suggests their potential for regulating this calcium-binding protein in melanoma. We have previously reported that HOXC11 can utilise the steroid receptor coactivator protein to drive tumour progression in breast cancer patients (McIlroy *et al*, 2010). In this study, we used combined immunohistochemical and immunofluorescence to assess expression of HOXC11 and its coactivator protein SRC-1 in benign nevi and malignant melanoma. We found high levels of expression and coassociation of HOXC11 and SRC-1 in malignant melanoma in comparison with benign nevi. Consistent with this, greater expression levels and coassociation of the transcription factor and coactivator were observed in the metastatic MeWo cells relative to the primary SkMel28 melanoma cell lines.

Recently, we and others have described a role for HOXC11 in the transcriptional regulation of S100beta in breast cancer and neuronal tissue (Zhang *et al*, 2007; McIlroy *et al*, 2010). The calcium-binding protein S100beta is one of the most widely researched biomarkers in melanoma and its serum expression has been shown to correlate with both disease stage and response to treatment (Mocellin *et al*, 2008). While there is substantial evidence to support transcriptional regulation of S100beta (Allore *et al*, 1990; Jiang *et al*, 1993; Zimmer *et al*, 1995), little is definitively known about its regulators in melanoma. Here we observed recruitment of HOXC11 to the S100beta promoter. Using a variety of molecular and cellular techniques, we established a role for both HOXC11 and SRC-1 in the regulation of S100beta, uncovering a new mechanism of S100beta regulation in melanoma.

Abnormalities in growth factor signalling pathways have an intrinsic role in melanoma disease progression. Systems that impede this signalling network, including those that target c-kit, raf and p-Src are at the forefront in the development of new therapeutic strategies to treat this disease. *In vitro* studies have shown that coactivator function of SRC-1 is mediated at least in part through p-Src (Shang and Brown, 2002; Shah and Rowan, 2005). We investigated whether inhibition of the Src kinase signalling network could have a role in disrupting the functional interaction between HOXC11 and SRC-1 in melanoma cells. The ATP-competitive dual Src/Abl inhibitor dasatinib inhibited HOXC11 and SRC-1 mediated S100beta production in malignant melanoma cells. Furthermore, in the metastatic melanoma cell line MeWo, treatment with dasatinib reduced cell migration. Dasatinib targets BCR/ABL and c-Kit, and is the most potent Src kinase inhibitor currently in clinical development. It has proven efficacy in the treatment of chronic myelogenous leukaemia (Talpaz *et al*, 2006). It is currently in trial for the treatment of advanced melanoma (http://www.clinicaltrial.gov). Though none of these trials have completed, interim reports suggest that as a single therapy, dasatinib has minimal activity in unselected patients (Kluger *et al*, 2010). Moreover, recent *in vitro* studies in the breast suggest that dasatinib may be more effective when combined with other targeted therapies rather than as a single agent in the treatment of solid tumours (Vallabhaneni *et al*, 2010).

In spite of recent advances in targeted therapy, no treatment has managed to significantly prolong overall survival in patients with advanced melanoma. Molecular interactions that link tumour progression to developmental biology provide the ideal milieu for a tumour to adapt and evade targeted therapies. Here, we describe a new HOX/SRC-1-S100beta signalling network that can be inhibited by dasatinib. Though initial promise for the use of dasatinib in the treatment of advanced melanoma may be tempered by early clinical data, targeting with new molecular markers has the potential to greatly increase the efficacy of this kinase inhibitor.

## Figures and Tables

**Table 1:** Associations of expression of HOXC11 and SRC-1 in malignant melanoma and benign nevi from patients using Fisher's exact test

	**HOXC11 *n* (%)**		**SRC-1 *n* (%)**	
	**Nuclear**	**Cytoplasmic**	**Nuclear**	**Cytoplasmic**
Melanoma (*n*=64)	34 (53.1)	19 (29.7)	30 (46.8)	21 (32.8)
Benign nevus (*n*=20)	1 (5)	8 (40)	3 (15)	6 (30)
*P*-value	<0.001	NS	0.017	NS

Abbreviation: NS=Not significant.

## References

[bib1] Allore RJ, Friend WC, O’Hanlon D, Neilson KM, Baumal R, Dunn RJ, Marks A (1990) Cloning and expression of the human S100 beta gene. J Biol Chem 265: 15537–15543 2394738

[bib2] Donato R, Sorci G, Riuzzi F, Arcuri C, Bianchi R, Brozzi F, Tubaro C, Giambanco I (2009) S100B's double life: intracellular regulator and extracellular signal. Biochim Biophys Acta 3: 1008–1022 10.1016/j.bbamcr.2008.11.009 19110011

[bib3] Gregory CW, He B, Johnson Jr RT, Ford OH, Mohler JL, French FS, Wilson EM (2001) A mechanism for androgen receptor-mediated prostate cancer recurrence after androgen deprivation therapy. Cancer Res 61: 4315–4319 11389051

[bib4] Jiang H, Shah S, Hilt DC (1993) Organization, sequence, and expression of the murine S100 beta gene. Transcriptional regulation by cell type-specific cis-acting regulatory elements. J Biol Chem 268: 20502–20511 8376406

[bib5] Kavanagh DO, McIlroy M, Myers E, Bane F, Crotty TB, McDermott E, Hill AD, Young LS (2010) The role of oestrogen receptor {alpha} in human thyroid cancer: contributions from coregulatory proteins and the tyrosine kinase receptor HER2. Endocr Relat Cancer 17: 255–264 20032008 10.1677/ERC-09-0216

[bib6] Kluger HM, Dudek AZ, McCann C, Ritacco J, Southard N, Jilaveanu LB (2010) A Phase 2 trial of Dasatinib in advanced melanoma. Cancer 117: 2202–2208 21523734 10.1002/cncr.25766 PMC3116034

[bib7] Maeda K, Hamada J, Takahashi Y, Tada M, Yamamoto Y, Sugihara T, Moriuchi T (2005) Altered expressions of HOX genes in human cutaneous malignant melanoma. Int J Cancer 114: 436–441 15551325 10.1002/ijc.20706

[bib8] McIlroy M, McCartan D, Early S, O Gaora P, Pennington S, Hill AD, Young LS (2010) Interaction of developmental transcription factor HOXC11 with steroid receptor coactivator SRC-1 mediates resistance to endocrine therapy in breast cancer. Cancer Res 70: 1585–1594 20145129 10.1158/0008-5472.CAN-09-3713

[bib9] Mocellin S, Zavagno G, Nitti D (2008) The prognostic value of serum S100beta in patients with cutaneous melanoma: a meta-analysis. Int J Cancer 123: 2370–2376 18752249 10.1002/ijc.23794

[bib10] Modi PK, Kanungo MS (2010) Age-dependent expression of S100b in the brain of mice. Cell Mol Neurobiol 30: 709–716 20099023 10.1007/s10571-009-9495-y PMC11498891

[bib11] Redmond AM, Bane FT, Stafford AT, McIlroy M, Dillon MF, Crotty TB, Hill AD, Young LS (2009) Coassociation of estrogen receptor and p160 proteins predicts resistance to endocrine treatment; SRC-1 is an independent predictor of breast cancer recurrence. Clin Cancer Res 15: 2098–2106 19276281 10.1158/1078-0432.CCR-08-1649

[bib12] Riker AI, Enkemann SA, Fodstad O, Liu S, Ren S, Morris C, Xi Y, Howell P, Metge B, Samant RS, Shevde LA, Li W, Eschrich S, Daud A, Ju J, Matta J (2008) The gene expression profiles of primary and metastatic melanoma yields a transition point of tumor progression and metastasis. BMC Med Genomics 1: 13 18442402 10.1186/1755-8794-1-13 PMC2408576

[bib13] Samuel S, Naora H (2005) Homeobox gene expression in cancer: insights from developmental regulation and deregulation. Eur J Cancer 41: 2428–2437 16199152 10.1016/j.ejca.2005.08.014

[bib14] Shah N, Sukumar S (2010) The Hox genes and their roles in oncogenesis. Nat Rev Cancer 10: 361–371 20357775 10.1038/nrc2826

[bib15] Shah YM, Rowan BG (2005) The Src kinase pathway promotes tamoxifen agonist action in Ishikawa endometrial cells through phosphorylation-dependent stabilization of estrogen receptor (alpha) promoter interaction and elevated steroid receptor coactivator 1 activity. Mol Endocrinol 19: 732–748 15528270 10.1210/me.2004-0298

[bib16] Shang Y, Brown M (2002) Molecular determinants for the tissue specificity of SERMs. Science 295: 2465–2468 11923541 10.1126/science.1068537

[bib17] Talpaz M, Shah NP, Kantarjian H, Donato N, Nicoll J, Paquette R, Cortes J, O’Brien S, Nicaise C, Bleickardt E, Blackwood-Chirchir MA, Iyer V, Chen TT, Huang F, Decillis AP, Sawyers CL (2006) Dasatinib in imatinib-resistant Philadelphia chromosome-positive leukemias. N Engl J Med 354(24): 2531–2541 16775234 10.1056/NEJMoa055229

[bib18] Vallabhaneni S, Nair BC, Cortez V, Challa R, Chakravarty D, Tekmal RR, Vadlamudi RK (2010) Significance of ER-Src axis in hormonal therapy resistance. Breast Cancer Res Treat; e-pub ahead of print 10.1007/s10549-010-1312-2 PMC3243930 21184269

[bib19] Wang S, Yuan Y, Liao L, Kuang SQ, Tien JC, O’Malley BW, Xu J (2009) Disruption of the SRC-1 gene in mice suppresses breast cancer metastasis without affecting primary tumor formation. Proc Natl Acad Sci USA 106: 151–156 19109434 10.1073/pnas.0808703105 PMC2629242

[bib20] Zhang X, Hamada J, Nishimoto A, Takahashi Y, Murai T, Tada M, Moriuchi T (2007) HOXC6 and HOXC11 increase transcription of S100beta gene in BrdU-induced *in vitro* differentiation of GOTO neuroblastoma cells into Schwannian cells. J Cell Mol Med 11: 299–306 17488478 10.1111/j.1582-4934.2007.00020.x PMC3822828

[bib21] Zimmer DB, Cornwall EH, Landar A, Song W (1995) The S100 protein family: history, function, and expression. Brain Res Bull 37: 417–429 7620916 10.1016/0361-9230(95)00040-2

